# Harshly Oxidized Activated Charcoal Enhances Protein Persulfidation with Implications for Neurodegeneration as Exemplified by Friedreich’s Ataxia

**DOI:** 10.3390/nano14242007

**Published:** 2024-12-13

**Authors:** Anh T. T. Vo, Uffaf Khan, Anton V. Liopo, Karthik Mouli, Kenneth R. Olson, Emily A. McHugh, James M. Tour, Madhavan Pooparayil Manoj, Paul J. Derry, Thomas A. Kent

**Affiliations:** 1 Center for Genomics and Precision Medicine, Institute of Bioscience and Technology, Texas A&M Health Science Center, Houston, TX 77030, USA; 2 Department of Chemistry, Rice University, Houston, TX 77005, USA; 3 Department of Physiology, Indiana University School of Medicine South Bend, South Bend, IN 46617, USA; 4 Department of Biological Sciences, University of Notre Dame, Notre Dame, IN 46556, USA; 5 Smalley-Curl Institute, Rice University, Houston, TX 77005, USA; 6 Rice Advanced Materials Institute, Rice University, Houston, TX 77005, USA; 7 The NanoCarbon Center, Rice University, Houston, TX 77005, USA; 8 School of Engineering Medicine, Texas A&M University, Houston, TX 77030, USA; 9 Stanley H. Appel Department of Neurology, Houston Methodist Hospital and Research Institute, Houston, TX 77030, USA

**Keywords:** nanozyme, persulfidation, Nrf2, Keap1, oxidized activated carbon nanoparticles

## Abstract

Harsh acid oxidation of activated charcoal transforms an insoluble carbon-rich source into water-soluble, disc structures of graphene decorated with multiple oxygen-containing functionalities. We term these pleiotropic nano-enzymes as “pleozymes”. A broad redox potential spans many crucial redox reactions including the oxidation of hydrogen sulfide (H_2_S) to polysulfides and thiosulfate, dismutation of the superoxide radical (O_2_^−^*), and oxidation of NADH to NAD^+^. The oxidation of H_2_S is predicted to enhance protein persulfidation—the attachment of sulfur to cysteine residues. Persulfidated proteins act as redox intermediates, and persulfidation protects proteins from irreversible oxidation and ubiquitination, providing an important means of signaling. Protein persulfidation is believed to decline in several neurological disorders and aging. Importantly, and consistent with the role of persulfidation in signaling, the master antioxidant transcription factor Nrf2 is regulated by Keap1’s persulfidation. Here, we demonstrate that pleozymes increased overall protein persulfidation in cells from apparently healthy individuals and from individuals with the mitochondrial protein mutation responsible for Friedreich’s ataxia. We further find that pleozymes specifically enhanced Keap1 persulfidation, with subsequent increased accumulation of Nrf2 and Nrf2’s antioxidant targets.

## 1. Introduction

Persulfidation is a post-translational protein modification of covalently linking a sulfur atom to a thiol group on cysteine. Over 30% of endogenous proteins exist in a persulfidated form [1,2], underscoring persulfidation as a protein modification of comparable significance to phosphorylation [3,4]. Persulfidation activates proteins and pathways responsible for mitochondrial biogenesis, ATP production, antioxidative responses, and neuroprotection [1,5,6,7].

Persulfidation exerts regulatory influence over numerous proteins and pathways. An example is the nuclear factor erythroid 2-related factor 2 (Nrf2)/Kelch-like ECH-associated protein 1 (Keap1) pathway that plays a central role in cytoprotection and sulfur metabolism [8,9]. Nrf2 is a master transcription factor regulating antioxidant and cytoprotective genes. Nrf2 predominantly binds to the Keap1 dimer, facilitating its ubiquitination. Numerous active cysteine residues on Keap1 serve as the site for persulfidation and subsequent inactivation [8,10,11,12], allowing Nrf2’s release and translocation to nucleus. Persulfidated Keap1 forms new disulfide bonds with another Keap1 (homodimer) or peptide (heterodimer), disrupting the configuration of Keap1-Nrf2 binding, resulting in a lower affinity of Keap1 for Nrf2 [13,14,15]. Existing Nrf2 stays bound to Keap1, while newly synthesized Nrf2 freely translocates to the nucleus [10], where Nrf2 activates the transcription of antioxidant genes such as *NQO1*, catalase (*CAT*), *GCLC*, and HO-1 [8,9].

A decline in persulfidation has been observed in aged brain and heart [4,16], neurological disorders such as Alzheimer’s disease [4,17,18], Parkinson’s disease [4,5,17,19,20], Down syndrome [21], Huntington’s disease [4], and other diseases with sulfur dysregulation such as Friedreich’s ataxia (FRDA) [22] Currently, the only approved therapy for the latter is SKYCLARYS^®^ (omaveloxolone), which increases Nrf2 levels by inhibiting its ubiquitination [23,24,25]. While improving features of the disease, there remains room for additional therapeutic approaches for FRDA and other neurodegenerative diseases [24,26]. Our group developed a novel therapy using nano-sized, metal-free oxidized carbon nanoparticles (CNPs) with pre-clinical therapeutic efficacy for disorders such as hemorrhagic stroke, traumatic brain injury, and features of accelerated aging seen in Down syndrome [27,28,29]. CNPs carry oxygen-containing moieties and exhibit enzyme-like properties, classifying them as nanozymes [30]. Starting from a medicinal-graded ingredient—coconut-derived activated charcoal—we performed a single-step synthesis with fuming nitric acid and high temperature to yield nano-sized, disc-shaped graphenic nanoparticles (OACs) with quinones, hydroxyls, and carboxyl groups. They are subsequently conjugated with poly(ethylene glycol), generating a functionalized material that we term PEGylated oxidized activated charcoal (PEG-OACs) [27].

In McHugh et al. [26], we identified an optimal set of reaction conditions to produce highly active yet also biologically compatible nanoparticles [26]. TEM imaging of the OACs prepared by oxidizing coconut-derived activated charcoal for 6 h at 100 °C revealed them to be approximately 2.5 nm in diameter. XPS analysis showed the OACs were primarily C=C/C-C (65.7%) content. The remainder consisted of C-O-C/C-O (11.1%), C=O (7.1%), and O-C=O (16.1%) content [26]. Cyclic voltammetry showed a strong reduction wave below 0 V, indicating the particles were good oxidizers [26]. PEGylation with amide-coupled poly(ethylene glycol) yields PEG-OACs. Following PEGylation, the nanoparticles are approximately 90% PEG by mass [26]. PEG-OACs are stable in water, PBS, or serum without additional agitation. Based on these characteristics, we determined that pleozymes carry a large quantity of quinoidal moieties, which may contribute to the electrochemical properties and potent capacity [27]. TEM images suggested that pleozymes co-localized with the mitochondria [31] and protected mitochondrial DNA and functions from hemin-induced oxidative stress [28]. Pleozymes administered intravenously or intra-peritoneally showed consistent therapeutic improvement in various forms of preclinical testing. In a traumatic brain injury rat model, pleozymes restored brain blood flow back to the basal blood flow [27]. A single injection of pleozymes at 80 min after brain injury recovered and maintained stable blood flow up to 300 min post-injury [27]. Pleozymes also showed improvement in brain injury gene response, infarct volume or hemisphere swelling in models of ischemic brain injury [32], intracerebral hemorrhage [28], and traumatic brain injury [27,33,34,35]. Pleozymes have not demonstrated overt toxicity and protect cells against H_2_O_2-_ and H_2_S-induced cell death [27,29].

What makes the nanozyme PEG-OACs interesting is that they have pleiotropic enzyme mimetic functions [29,36,37]; therefore, we term them “pleozymes”. We hypothesized that pleozymes promote the oxidation of H_2_S to polysulfides by using the quinone moieties as a catalytic platform to transfer electrons between H_2_S to other electron acceptors, suggesting that pleozymes can participate in sulfur metabolism [29]. Protein persulfidation is an important intermediate in polysulfide metabolism and is a mechanism of interest in this study. We hypothesize that pleozymes promote a protective response [28,33,38,39] to counteract elevated H_2_S levels [29] by facilitating protein persulfidation, particularly that of Keap1, leading to increased levels of Nrf2 and its downstream antioxidant gene targets.

## 2. Methods

### 2.1. Cell Lines

Mouse brain endothelial cells (b.End3) were cultured in Dulbecco’s modified Eagle’s media (DMEM, high glucose and sodium pyruvate) supplemented with 10% fetal bovine serum and 1% penicillin–streptomycin. Cells were grown at 37 °C and 5% CO_2_. Cells were allowed to grow until reaching 80–90%, then were trypsinized and re-seeded at a 1:4 ratio.

Skin fibroblasts from apparently healthy individuals (AHI) were obtained from the Coriell Institute of Medical Research (Camden, NJ, USA). Cells were grown in minimum essential medium (MEM) (Gibco) with penicillin–streptomycin and 15% fetal bovine serum (Avantor-non heated). These control fibroblasts were age-matched to the fibroblasts from Friedreich’s ataxia (FRDA) patients, provided by Dr. Marek Napierala, UT Southwestern part of Friedreich’s Ataxia Research Alliance Cell Line Repository (FACLR). Two male FRDA skin fibroblasts (F188, F4675) and two female (F68, F4627) FRDA skin fibroblasts were used for the FRDA study. The samples were collected by punch skin biopsy samples from participants with approval from the Children’s Hospital of Philadelphia (CHOP) and University of Alabama (UAB) Institutional Review Boards (CHOP IRB #10-007864; UAB IRB #N131204003) (Friedreich’s Ataxia Cell Line Repository (FACLR)) [40,41,42].

### 2.2. PEG-OAC Synthesis

PEG-OACs (6h fuming nitric acid) was prepared as published [27,29] and reiterated here. Powdered medical-grade coconut-shell activated charcoal (AC) was mixed with fuming (90%) nitric acid (HNO_3_) at 100 °C for six hours continuously. The mixture was then allowed to cool to room temperature and quenched with ice, which was made from deionized water. The mixture was filtered through dialysis with continuous water flow over 7 daysusing a regenerated cellulose membrane with a 1 kDa molecular weight threshold.. The filtered mixture was further purified through a 0.22 µm poly(ethersulfone) (PES) membrane and dried by lyophilization. The products were termed oxidized activated charcoals (OACs). OACs were resuspended in dimethylformamide (DMF) and subsequently mixed with 5 kDa methoxy-PEG-amine in a vessel. The mixture was sonicated in water for 20 min, then stirred continuously at room temperature for 48 h. The mixture was then dialyzed using a Float-A-Lyzer G2 dialysis device in a water bath for 48 h at room temperature. The water bath was changed every 6–8 h within the 48 h period. After the reaction stopped, the products were purified using a 0.22 µm PES membrane. The purified products were termed PEG-OACs, or pleozymes. We determined the concentration of the nanoparticle solution by first carefully massing lyophilized OACs and dissolving them into a known volume. A calibration curve was created using this solution to calculate the extinction coefficient at 700 nm. Afterwards, the concentration of PEG-OACs was measured using an Agilent 8453 UV–VIS Diode Array Detector Spectrophotometer. PEG-OACs subsequently were diluted to 160 µg/mL in sterile PBS, and the final concentration of 4 µg/mL was used in each experiment.

### 2.3. Protein Persulfidation Labeling Using the Dimedone Switch Method

The persulfidation protocol was adapted from Zivanovic et al., 2020, with the following adjustments [20]. All steps were performed in the dark. Day 1: bEnd.3 cells were seeded on 10 cm Petri dishes (Thermo Fisher, Waltham, MA, USA, Nunc™ EasYDish™ Dishes, cat. 150464) at a concentration of 50,000 cells/mL. Cells were grown for 2 days, 80–90% confluency was reached, and they were subsequently treated with effectors for 1 h. After treatments, the medium was gently discarded using an aspirator. Cells were washed twice with 2 mL cold phosphate-buffered saline, which was added to the Petri dish using a 1000 µL pipet and subsequently removed by the aspirator. Cells were mechanically lysed with disposable plastic scrapers in 1 mL of HEN lysis buffer, supplemented with 5 mM NBD-Cl (in DMSO) and 1x protease inhibitor cocktail (Thermo Fisher, Cat. # 78429) immediately before use. Stock of HEN lysis buffer was stored at −20 °C and thawed at 4 °C. Scraped cells were transferred to a 15 mL tube and subsequently lysed with 18G syringes 7 times. The tube was vortexed and incubated at 37 °C for 30 min, protected from light. After 30 min, cold methanol and cold chloroform were added to the lysate following the ratio of 4:4:1, *v*/*v*/*v* (lysate/methanol/chloroform).

The tube was centrifuged at 14,000× *g*, 15 min, 4 °C (NuAire Multi-Application Centrifuge using the RA24-2 rotor; NuAire, Minneapolis, MN, USA,). A protein pellet was formed between the organic and aqueous layers, which were aspirated gently. Next, the pellet was washed with cold water/methanol/chloroform (4/4/1, *v*/*v*/*v*). Methanol was added to the tube first. And the pellet was transferred to a new microcentrifuge tube. Cold water and chloroform were added to the microcentrifuge tube to achieve the ratio of 4:4:1, *v*/*v*/*v* (water/methanol/chloroform). The microcentrifuge tube was centrifuged at 14000× *g*, 15 min, 4 °C, and the supernatant was aspirated again. Then, the pellet was rinsed with 1 mL cold methanol twice by inverting and air-dried.

The dried pellet was re-dissolved by soaking the pellet in 10 µL of 20% SDS in H_2_O for 30 min to soften the pellet. Subsequently, 90 µL of 50 mM HEPES (final conc. SDS is 2%)* was added to the pellet, the mixture was vortexed, and then we waited until the pellet was re-dissolved. Lysate was stored at −20 °C in the dark overnight or for a few weeks.

Day 2: The pellet was thawed at 4 °C and subsequently at room temperature. Protein concentration was measured using the Bradford assay. We adjusted the lysate concentration to 3 mg/mL by adding more HEPES with SDS (final conc. 2%) to the lysate. DAz-2:Cy-5 click mix was stored at −80 °C and thawed at room temperature while continuously being mixed for at least 15 min before use. DAz-2:Cy-5 click mix was added to the 3 mg/mL lysate to a final concentration of 25 µM DAz-2:Cy5 click mix and mixed well. The mixture was incubated at 37 °C for 30 min and protected from light. The lysate was subsequently washed by lysate/methanol/chloroform, followed by water/methanol/chloroform and methanol twice. The pellets were redissolved in HEPES with SDS (final conc. 2%). Steps are described in the day 1 protocol. Lysate was stored at −20 °C in the dark, for a maximum of a week, because the signal from the DAz-2:Cy5 click mix would gradually decrease.

Day 3: The protein concentration was measured using the Bradford assay and adjusted to 2 mg/mL with HEPES with SDS (final conc. 2%). Lysate was used for the SDS-PAGE assay on the same day.

### 2.4. Protein Quantification

Protein concentration was determined using the Pierce™ Detergent Compatible Bradford Assay Kit (Thermo Fisher, Waltham, MA, USA; catalog 23246) and measured in the CLARIOstar BMG Labtech microplate reader (BMG Labtech GmbH, Ortenburg, Germany).

### 2.5. SDS-PAGE

Lysates were adjusted to have the same concentration across samples. The following steps were performed in a chemical hood to avoid exposure to β-mercaptoethanol (Millipore Sigma, Burlington, MA, USA; catalog M6250). Three equivalents of the lysate were mixed with one equivalent of 4x Bolt™ LDS Sample Buffer (Thermo Fisher, Waltham, MA, USA; catalog B0007) supplemented with 10% β-mercaptoethanol (Millipore Sigma, Burlington, MA, USA; catalog M6250). The tube was vortexed, heated at 75 °C for 15 min, and briefly spun down so that precipitate was collected at the bottom of the Eppendorf tube. The 10% SDS-PAGE gel (Thermo Scientific, Waltham, MA, USA; cat. NW00102BOX) was used. It was important to fill all wells; we filled empty wells with Bolt™ LDS Sample Buffer. We ran the gel at 60 mA, 150 V max, for an hour in the dark.

### 2.6. Gel Imaging

We disassembled the SDS-PAGE gel and transferred the gel to a container with mqH2O, and then covered it in foil. The fluorescence was measured on ProteinSimple: FluorChem M Imaging Systems (Minneapolis, Minnesota). The persulfidation signal was recorded at 632 nm, under a multi-fluorescence red channel (filter: far red, time: auto). The NBD-Cl signal was recorded at 475 nm, under a multi-fluorescence blue channel (filter: green, time: auto).

### 2.7. Western Blot

Cell extraction was performed using Cell Lysis Buffer and the provided protocol (Thermo Fisher, Waltham, MA, USA; catalog #FNN0011). Proteins were quantified using the Bradford assay (Thermo Fisher, Waltham, MA, USA; catalog #1863028). The same amounts of proteins of each lysate (20–30 µg) were loaded on an SDS-PAGE gel with 1x Bolt™ Sample Reducing Agent (Thermo Fisher, Waltham, MA, USA; catalog B0009) and 1x Bolt™ LDS Sample Buffer (Thermo Fisher, Waltham, MA, USA; catalog B0007). Membrane transfer was performed overnight in a cold room and blocked with 5% dry milk/TBST the next day. Subsequently, the membrane was incubated with primary antibodies overnight and secondary antibodies for an hour in a blocking solution. Signals were developed with ECL (Thermo Fisher, Waltham, MA, USA; catalog #A43840) and visualized using aChemiDoc^TM^ Imaging System (Version 2.4.0.03, Bio-Rad Laboratories, Hercules, CA, USA).

### 2.8. Redox Western Blot

The protocol was similar to the common Western blot protocol, with exceptions listed below. In each step, such as washing and lysing samples, we supplemented PBS and lysis buffer with 40 mM N-ethylmaleimide (NEM, Thermo Fisher, Waltham, MA, USA, catalog #23030). Lysates were diluted in non-reducing condition with 1x Bolt™ LDS Sample Buffer (Thermo Fisher, Waltham, MA, USA, catalog B0007) and without reducing agents. Other steps are identical to the Western blot’s protocol.

### 2.9. Protein Aggregation

Reaction mixtures of 40 µM bovine erythrocyte SOD1 (Sigma-Aldrich, St. Louis, MO, USA; Cat. # S7571), 40 μM thioflavin T (Cayman Chemical, Ann Arbor, MI, USA; Cat. #32553), 150 μM disodium sulfide nonahydrate (Sigma-Aldrich, St. Louis, MO, USA; Cat. # 431648), and 150 μM hydrogen peroxide (Sigma-Aldrich, St. Louis, MO, USA; Cat. # H1009) were prepared in phosphate-buffered saline in a procedure adapted from Malik et al. [43]. A total of 100 µL of each reaction mixture was loaded per well in a 96-well plate. Fluorescence readings were measured (λEx./λEm.: 420/480 nm) every 15 min over a 72 h interval using a BMG CLARIOstar Microplate Reader (BMG Labtech GmBH, Ortenburg, Germany) under shaking at 300 rpm at 37 °C.

### 2.10. Quantification and Statistical Analysis

Persulfidation gel images were analyzed by using the polygon selection tool in ImageJ. The region-of-interest (ROI) was drawn from 140 kDa (the highest protein ladder lane) to 40 kDa (the 5th protein ladder lane). The raw integrated intensity was measured in each channel, and the ratio of 632/488 nm signals was calculated in Excel. The analysis for brain endothelial bEnd.3 cells on SDS-PAGE gel was a paired, two-tailed t-test. The analysis for Keap1 persulfidation was one-way ANOVA followed by Tukey’s multiple comparisons test. The plotting graphs and analysis were performed using GraphPad Prism version 10.0.0 for Windows. All statistical analyses were tested against null hypotheses, α = 0.05.

## 3. Results

### 3.1. PEG-OACs Induced Protein Persulfidation

**Figure 1:**
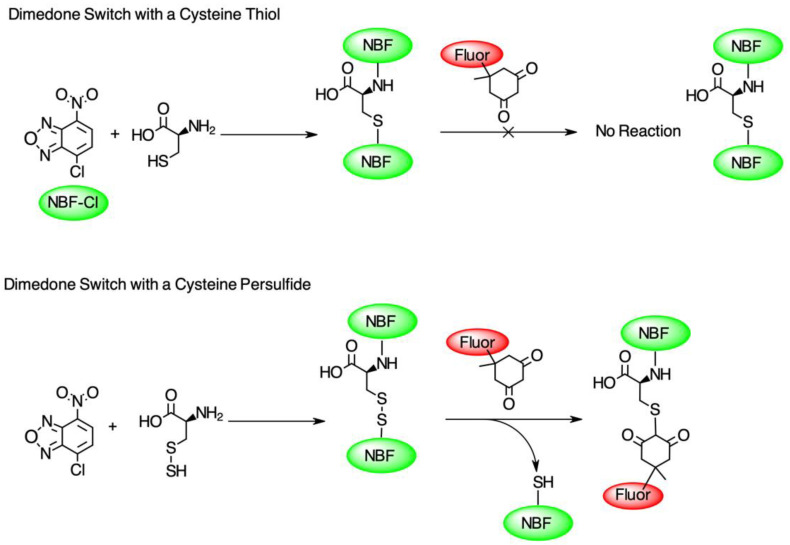
The diagram of the dimedone-switch method to detect persulfidation. NBF-Cl forms a bond with a sulfur atom in thiol (-SH), persulfide (-SSH), and nitrogen atoms in the amino group (-NH_2_) and tags the protein with a green fluorescence (first reactions in top and bottom panels). A dimedone switch selectively replaces the NBF-binding on persulfide and labels persulfide with a red fluorescence (second reaction in bottom panel). The dimedone switch cannot displace the NBF-binding on the thiol or amino group (second reaction in top panel). Figure adapted from Živanović et al., 2020 [20].

To test the hypothesis that pleozymes induced protein persulfidation, we adopted a two-step persulfidation labeling method from Dr. Živanović’s group [20]. 4-chloro-7-nitrobenzofurazan (NBF-Cl) reacts with the sulfur atom in persulfides (-SSH), thiols (-SH), sulfenic acids (-SO_3_^−^), and the nitrogen atom in amino groups (-NH_2_), generating green fluorescent adducts. The second step of labeling involves a dimedone-based reagent, which selectively replaces the NBF fluorophore in the labeled persulfide adducts and produces a Cy5-labeled persulfide adduct. The persulfidation levels were quantified as the ratio of fluorescence signals at Cy5 and 488 nm excitation wavelengths (Figure 1).

Mouse brain endothelial (bEnd.3) cells were treated with the H_2_S donor GYY4137 to generate intracellular H_2_S to test the effect of excess H_2_S on protein persulfidation. Persulfidation, like other post-translational protein modifications, occurs spontaneously and as early as 30 min through enzymatic activities of cystathionine β-synthase (CBS), cystathionine γ-lyase (CSE), 3-mercaptopyruvate sulfurtransferase (MPST), and thiosulfate sulfurtransferase (TST), among others [44,45,46]. Persulfidation reaches peak levels after 1 hourbefore it either plateaus or declines [44]. Therefore, we measured persulfidation 1 to 1.5 h after treatment.

**Figure 2:**
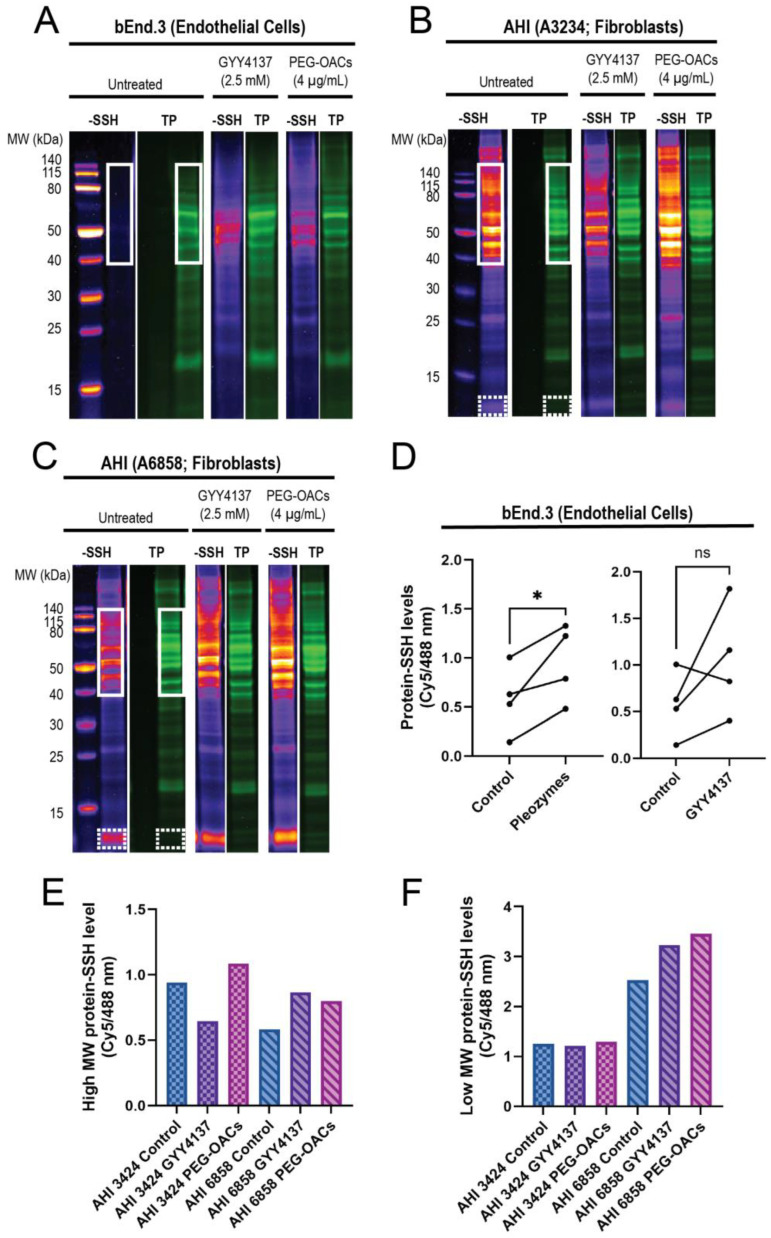
Pleozymes induced global protein persulfidation in endothelial cells and fibroblasts, with a focus on high- and low-molecular-weight proteins. (**A**) Persulfidation signals relative to total protein increased with pleozyme treatment at therapeutically relevant dosages of 4 µg/mL versus untreated control in cultured brain endothelial (bEnd.3) cells. The high-molecular-weight (MW) proteins from 40 to 140 kDa (white box) expressed less persulfidation in the control and enriched persulfidation/total protein with pleozyme treatment and were less consistent from the H_2_S donor, GYY4137. One representative gel image/experiment from N = 4 independent experiments. (**B**,**C**) Pleozymes also increased persulfidation/total protein ratio in fibroblasts from apparently healthy individuals (AHI). Fibroblasts exhibited two persulfidation-rich regions with pleozymes: high MW from 40 to 140 kDa (white box) and low molecular weight from 12 to 13 kDa (white, dash box). (**D**) The persulfidation-rich region/total protein region in endothelial cells quantified with N = 4 independent experiments was significantly different for the PEG-OAC pleozymes. Student’s *t*-test, paired, two-tailed, * represented *p* < 0.05. Individual values for high MW and low MW persulfidation-rich region/total protein region quantification are shown in (**E**,**F**), including the H_2_S donor, GYY41237, whose effects were less consistent than the pleozymes.

In endothelial cells, the proteins in the 40–140 kDa range exhibited minimal persulfidation in the control group, with higher persulfidation levels observed following treatments with GYY4137 and pleozymes (Figure 2A,D). Proteins outside this molecular weight range displayed minimal persulfidation in the control group and a non-significant trend for persulfidation with GYY4137 and pleozyme treatment. Within the 40–140 kDa region, pleozymes increased persulfidation two-fold after one-hour to one-and-a-half hour of treatment, while GYY4137, a slow-releasing of H_2_S donor, induced nearly a three-fold increase compared to the control group after two hours of incubation, although it was not as consistent as the pleozymes. Overall, pleozyme treatment significantly increased cellular persulfidaton levels (Figure 2A,D).

Interestingly, we discovered that the high-molecular-weight region exhibited elevated persulfidation in fibroblasts (Figure 2B,C), spanning from 40 kDa and above. Fibroblasts from two apparently healthy individuals (AHI), AHI3424 and AHI6858, also exhibited high levels of persulfidation in the low-molecular-weight region (12–13 kDa) (Figure 2B,C). These findings suggest that persulfidation and the susceptibility of some proteins to persulfidation are regulated in a cell-type-dependent manner. The effect of pleozymes on protein persulfidation was also evident in fibroblasts, inducing persulfidation in both high-molecular-weight (Figure 2E) and low-molecular-weight regions (Figure 2F). GYY4137, however, appeared to increase persulfidation in AHI6858 and not AHI3424. We are currently sequencing peptides to identify the low-molecular-weight persulfidated proteins. A potential consequence of pleozyme-induced persulfidation is the formation of sulfur bridges that may facilitate pathological protein aggregation. When superoxide dismutase 1 (SOD1) was incubated with pleozymes and excess sulfide ions, no notable changes in fluorescence of the protein aggregation marker thioflavin T were observed (Figure S1). Our observations therefore suggest that pleozymes may not induce the pathological aggregation of proteins in the presence of sulfide donors, though further experiments on a wider range of proteins would be necessary.

### 3.2. Pleozymes Induced the Expressions of Nrf2 When Intracellular H_2_S Pool Increased

**Figure 3:**
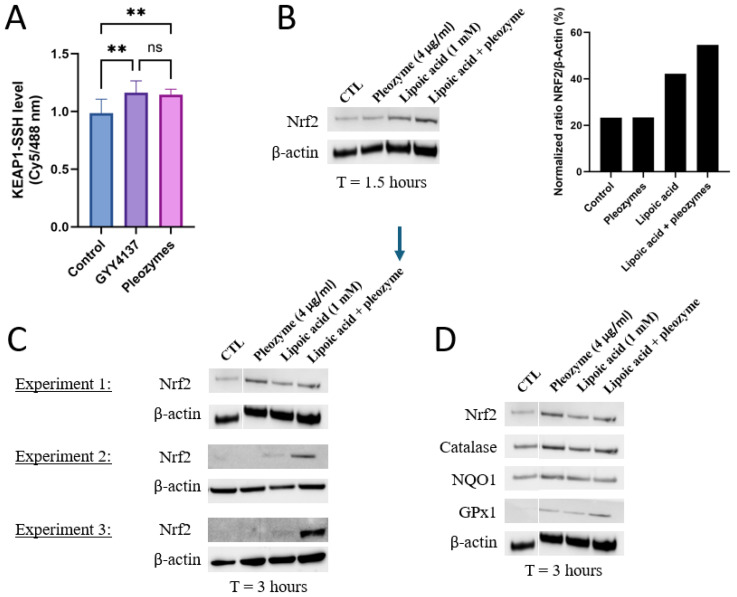
Pleozymes increased Keap1 persulfidation and induced the expression of Nrf2 and its antioxidant target genes. (**A**) Pleozymes increased KEAP1 persulfidation in brain endothelial (bEnd.3) cells at t = 1 h. N = 4 technical replicates. One-way ANOVA followed by Tukey’s multiple comparisons test, ** represented *p* < 0.01, ns represented not significant. Error bars represented standard deviation from 4 technical replicates. (**B**) Pleozymes induced Nrf2 with the presence of lipoic acid, an H_2_S donor. (**C**) Co-treating pleozymes with lipoic acid prolonged the pleozyme-induced Nrf2 accumulation (3 h). This was seen in all 3 independent experiments. (**D**) The expression of Nrf2 targets increased after treatment with pleozymes and/or lipoic acid.

Given that pleozymes induced persulfidation across a broad range of proteins from 40 kDa to 140 kDa, we hypothesized that pleozymes could induce persulfidation of Keap1, which has a molecular weight of 70 kDa [13]. Keap1, with its numerous active cysteine residues [8,10,11,12], serves as the prime target for persulfidation. To further determine whether cysteines form persulfide bonds, we performed an antibody-array-like assay using Keap1 antibodies and the persulfidation-labeled cell lysates, with the protocol adapted from Živanović et al. [20] We found that after one hour of treatment, pleozymes induced Keap1 persulfidation to a similar level of the positive control GYY4137, suggesting that pleozymes trigger Keap1 inactivation (Figure 3A).

Nrf2, a master regulator of the antioxidant response, is constitutively ubiquitinated by Keap1, resulting in its proteasome-mediated destruction, and as a result, the relative half-life of Nrf2 is approximately 20 min depending on the cell type [8,47]. When Keap1 is inactivated by persulfidation, Nrf2 is released to rapidly respond to stimuli and initiate the transcription for antioxidant genes. Because the Keap1 persulfidation was observed at t = 1 h after pleozyme treatment, the time point to investigate Nrf2 accumulation was chosen at 1.5 h.

We first investigated whether pleozymes could induce Nrf2 abundance within a shorter time interval(1.5 h). Nrf2’s stability is tightly regulated at the transcriptional and post-transcriptional levels depending on baseline oxidative status, making Nrf2 levels difficult to measure accurately and consistently [48,49,50]. We also stimulated cells with a positive control- lipoic acid, an H_2_S donor- which has been proposed to persulfidate Keap1 to increase Nrf2 release [51,52,53]. At 1.5 h, Nrf2 protein expression was induced with lipoic acidbut not pleozymes. However, pleozymes appeared to increase Nrf2 with lipoic acid co-treatment (Figure 3B).

We then investigated Nrf2 abundance at a longer period of 3 h. In two out of three experiments, lipoic acid induced Nrf2 expression at 3 h. In the third experiment, lipoic acid did not induce Nrf2. In one of these experiments, pleozymes alone induced Nrf2 expression more than lipoic acid. In the other two experiments, pleozymes did not induce Nrf2 individually, but co-treatment of pleozymes with lipoic acid induced Nrf2 expression consistently across three experiments at 3 h (Figure 3B,C). The expressions of Nrf2 targets, such as catalase, NAD(P)H dehydrogenase quinone 1 (NQO1), and glutathione peroxidase 1 (GPX1), followed the trends on Nrf2 abundance (Figure 3D). The results suggest that increased H_2_S by lipoic acid potentially fueled the catalytic reaction of the pleozymes, allowing pleozymes to catalytically convert H_2_S to polysulfide [29], then leading to other sulfide sulfur compounds such as persulfidation and inducing Nrf2 and antioxidant expression.

To explain the discrepancy in the effects of pleozymes on Keap1 persulfidation and Nrf2 abundance, we investigated another Keap1 oxidation modification via its disulfide bonds. Increases in Keap1 disulfide oxidation are linked to increased Nrf2 abundance [14]. Cysteine residues in Keap1 can form oxidized intermolecular (OxIR) disulfide bonds to form homo- and heterodimers with proteins. Keap1 can also form oxidized intramolecular (OxIM) disulfide bonds between two cysteine residues within a single Keap1 molecule, especially when the two cysteines are distant in the Keap1 sequence [14].

To visualize all OxIR and OxIM oxidative forms of Keap1 by Western blot, disulfide linkages between their cysteine residues were preserved by adding N-ethylmaleimide (NEM) during each step of the lysate preparation, while excluding reducing agents during the heat denaturation step. This method is referred to as redox SDS-PAGE [13,14,15]. Under these conditions, some Keap1 remained in its monomeric form and is expected to appear at 70 kDa, denoted as reduced Keap1 [14]. The OxIR and OxIM forms have a higher molecular weight and migrate more slowly than monomeric Keap1, resulting in multiple Keap1 bands.

**Figure 4:**
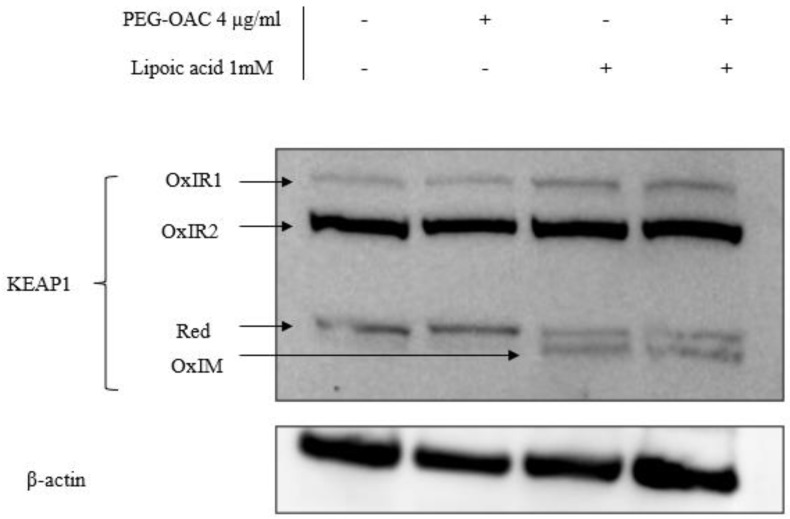
Co-treatment of pleozymes and lipoic acid, a H_2_S donor, induced the formation of the disulfide intramolecular (OxIM) bond of Keap1 after 3 h. Brain endothelial (bEnd.3) cells were treated with pleozymes and/or lipoic acid for three hours, following which cell lysates were collected and prepared for a redox Western blot to visualize oxidative modifications of Keap1. Keap1 existed in its reduced form (reduced band) and two oxidative forms (OxIR1 and OxIR2 bands with oxidized intermolecular disulfide bonds) at basal conditions. In the pleozyme-treated group, pleozymes maintained Keap1 oxidative expressions similarly to the control group. Exposure to lipoic acid, a H_2_S donor, induced the appearance of the oxidized intramolecular (OxIM) disulfide bonds and decreased the intensity of the reduced band, regardless of pleozymes treatment.

We adapted the protocol from previous studies [14,15] and studied the oxidative modification of Keap1 in the presence of excess H_2_S and pleozyme treatment. In the control sample, Keap1 was detected in three forms: one reduced and two OxIR forms, designated as OxIR1 and OxIR2. After three hours of pleozyme treatment, Keap1 expression remained consistent across these three forms, similar to the basal condition. The extended incubation time for this experiment was three times longer than the persulfidation experiments, which may explain unchanged oxidized forms after pleozyme treatment. In contrast, treatment with lipoic acid induced the expression of the OxIM band and decreased the intensity of the reduced band, while the OxIR1 and OxIR2 bands remained unaffected. This suggests that some monomeric Keap1 was oxidized to form OxIM Keap1. The results were consistent across lipoic acid treatment and with pleozyme addition, suggesting that lipoic acid had a more dominant effect on changing Keap1 oxidative forms, particularly inducing intramolecular (OxIM) disulfide bond formation compared to pleozymes after three hours (Figure 4). It is likely that the formation of OxIM Keap1 is a more robust indicator for Nrf2 abundance than Keap1 persulfidation. The mechanism by which lipoic acid oxidizes Keap1 is unclear and could involve generating oxidized forms of thioredoxin involved in the reduction of lipoic acid to dihydrolipoic acid [54]. Further time- and dose-dependent experiments are necessary to elucidate Keap1’s oxidation patterns under various pathological conditions.

### 3.3. Pleozymes Increased Protein Persulfidation in Patient-Derived FRDA Skin Fibroblasts

**Figure 5:**
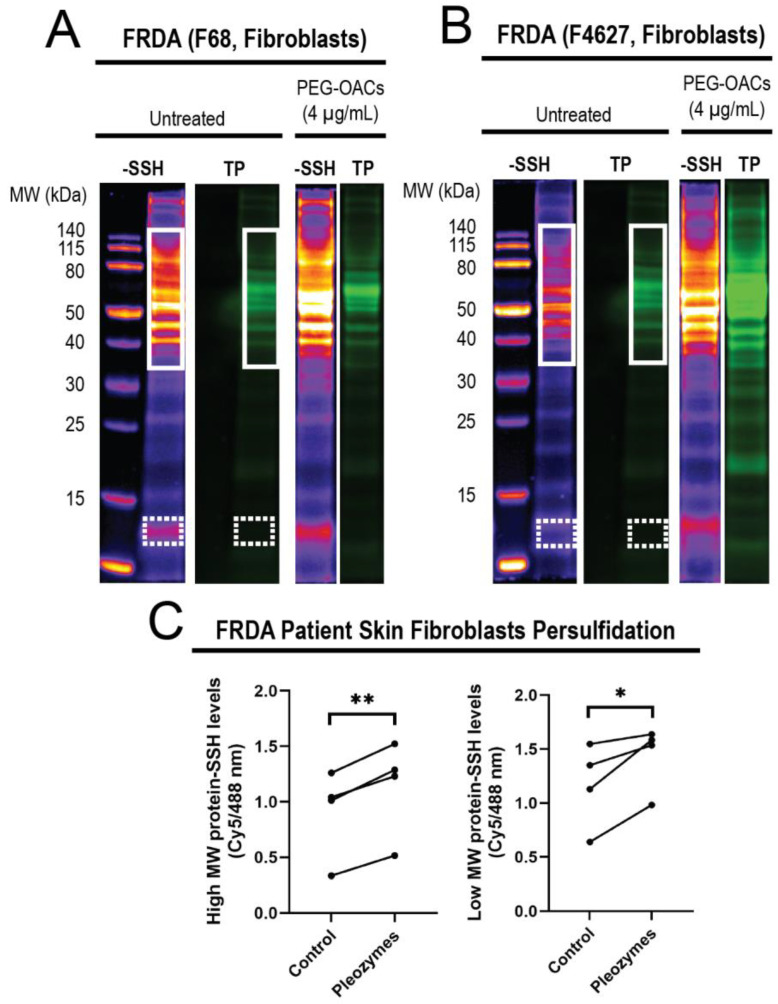
Treatment of FRDA skin fibroblasts (N = 4) with pleozymes (t = 1 h) significantly increased the persulfidation of both higher- and lower-molecular-weight proteins. In skin fibroblasts, cultured cells derived from Friedreich’s ataxia individuals (FRDA), persulfidation signals relative to total protein increased with pleozyme treatment at therapeutically relevant dosages of 4 µg/mL versus untreated (N = 4 biological replicates, 2 females and 2 males). Two representative examples (**A**,**B**) demonstrated enriched persulfidation in high-molecular-weight (MW) proteins from 40 to 140 kDa (white box) and lower-molecular-weight proteins from 12 to 13 kDa (dashed white box) in these FRDA fibroblasts compared to the untreated group. (**C**) Quantification of the intensity ratio of the -SSH (persulfidation) intensities/total protein intensities. Statistical analysis between untreated and treated groups was performed with the paired *t*-test, * represented *p* < 0.05, and ** represented *p* < 0.01.

After observing that pleozymes enhanced persulfidation in cells from apparently healthy individuals, we examined whether a similar enhancement occurs in cells obtained from individuals with Friedreich’s ataxia (FRDA), a neurodegenerative disease. FRDA is an autosomal recessive trinucleotide repeat disorder which affects approximately 1 in 50,000 live births in the USA, with almost all patients developing cardiomyopathy in addition to ataxia [55]. Individuals with FRDA exhibit over 200 GAA repeats in the *FXN* gene, in contrast to healthy individuals who have fewer than 40 GAA repeats [56]. *FXN* encodes for the protein frataxin, a chaperone that facilitates the biogenesis of Fe-S clusters (ISC), crucial for the function of mitochondrial respiratory chain proteins. As ISC formation depends on the formation of an intermediate persulfide by mitochondrial cysteine desulfurase (NFS1) [47], we hypothesized that decreased levels of frataxin in FRDA may relate to a persulfidation deficiency. We investigated levels of global protein persulfidation in FRDA-patient-derived skin fibroblasts after pleozyme treatment. After one hour treatment with pleozymes, we observed an increase in persulfidation in the treated group versus untreated control, which occurred both in high-molecular-weight proteins (*p* < 0.01) and lower-molecular-weight proteins (*p* < 0.05) (Figure 5).

## 4. Discussion

bEnd.3 cells have been used as a screening model system for studying the blood–brain barrier in the context of brain injuries and aging-related disorders, such as Parkinson’s disease and intracerebral hemorrhage [57,58,59], to validate pleozymes’ mechanisms of action in bEnd.3 cells. Additionally, skin fibroblasts are a frequently utilized model to investigate genetic disorders, so results obtained here may provide a basis to investigate other disorders [60].

Protein persulfidation protects proteins against irreversible excessive oxidation [20], increases sirtuin (SIRT) activities, promotes ATP generation and lactate dehydrogenase (LDH) activity to convert lactate to pyruvate, and stimulates mitochondrial biogenesis [6]. In neurodegenerative diseases like Alzheimer’s disease (AD), Parkinson’s disease (PD), and Friedreich’s ataxia (FRDA), recent research suggests that persulfidation is a common factor regulating detrimental disease mechanisms- therefore, interventions which increase persulfidation hold promise as potential therapies [17,18,19,20,21,22,61,62,63]. For instance, a feature of AD molecular pathophysiology is the hyperphosphorylation of tau protein and subsequent aggregation. Hyperphosphorylation of tau is inhibited by the persulfidation-mediated inactivation of glycogen synthase kinase (GSK3β). In healthy individuals, cystathionine γ-lyase (CTH) interacts with tau to produce H_2_S, contributing to the persulfidation and inactivation of GSK3β. In aging and AD, the loss in CTH expression results in lower GSK3β persulfidation and higher tau hyperphosphorylation and aggregation [17,18].

Similarly, protein aggregation is associated with functional decline in PD patients. Parkin, an E3 ubiquitin ligase, requires persulfidation for activation to ubiquitinate target proteins [6]. Additionally, parkin availability depends on the persulfidation of ubiquitin specific peptidase 8 (USP8), a deubiquitinase, to prevent parkin degradation, promote parkin migration to damaged mitochondria, and initiate mitophagy [6]. Overall, the lack of parkin and its persulfidation leads to protein aggregation, a hallmark of PD patients [17]. Another protein associated with PD is parkinsonism-associated deglycase 7 (PARK7, also formerly known as DJ-1), which has been shown to increase its activity through persulfidation [19,20].

Persulfidation and Nrf2 are intricately controlled mechanisms within biological systems. Their stringent regulations suggest their significant roles in influencing the progression of diverse diseases. As such, these factors have become focal points in research, as targeting them holds potential for developing novel therapeutic strategies aimed at mitigating or treating these conditions [8,64].

Persulfide prodrugs demonstrate enhanced pharmacological effects [65], suggesting that persulfidation has a therapeutic potential and deserves further investigation for treatment strategies. Nrf2 inducers have demonstrated therapeutic efficacy across a spectrum of aging-related and inherited neurological disorders; one Nrf2 drug has received FDA approval [23]. These drugs have demonstrated benefits in acute and chronic pre-clinical models such as hemorrhagic stroke [66,67], ischemic stroke [68,69], multiple sclerosis [70], and clinically in FRDA [71]. Their success in pre-clinical studies and clinical trials suggests Nrf2 as a promising target for a range of neurodegenerative disorders, offering hope for improved management and outcomes of these challenging diseases.

As discussed above, decreased persulfidation has been shown in many neurodegenerative diseases. However, although it has been shown that frataxin regulates the Fe-S cluster formation and persulfidation is an important step in that biogenesis, it has not been investigated in the context of overall persulfidation in FRDA-patient-derived samples. Our work here shows here for the first time with pro-persulfidation therapeutic-like pleozymes, we can increase the level of persulfidated proteins in FRDA fibroblasts. This increase may potentially help these cells to cope with impaired mitochondrial function, and it requires further investigation. Our group has recently reported on pleozymes’ ability to improve glycolytic and mitochondrial energy metabolism in cell and animal models of traumatic brain injury with hemorrhage, and it is possible that enhanced persulfidation synergizes with the pro-energetic functions of pleozymes to augment energy metabolism in FRDA [35].

Pleozyme therapy has the capacity to regulate both persulfidation and Nrf2 and provide cellular protection against mitochondrial and other toxins, such as excess H_2_S [29], hemin [28], and H_2_O_2_ [27]. Recently, our group discovered that pleozymes induce polysulfide and thiosulfate formation in the elevated H_2_S condition as a potential pathway to alleviate H_2_S toxicity in cells from individuals with Down syndrome [29]. We expected that polysulfide would mostly be excreted to biological fluids [72,73] and hypothesized that polysulfide would be metabolized into intermediates such as persulfides [74] and other sulfur-containing compounds as part of H_2_S detoxification. Our data suggest that pleozymes activate the antioxidant defense through protein persulfidation and Nrf2-regulated antioxidant genes, especially in the situation of increased H_2_S, which we accomplished through addition of lipoic acid. Interestingly, the effect of pleozymes on persulfidation did not significantly affect the persulfidation of GAPDH, an important glycolytic enzyme for energy metabolism. We did not observe a significant increase in GAPDH persulfidation (Figure S2). The persulfidation effects might be protein specific, time related, or other factors. Nevertheless, our results suggest that pleozymes have the potential to become a metabolic therapy to treat patients with sulfur dysregulation pathologies.

## 5. Conclusions

Our previous studies demonstrated that pleozymes catalyze the oxidation of H_2_S to polysulfide [29], thereby promoting the formation of persulfidated cysteines, as shown here **(**Figure 2). Keap1 persulfidation and inactivation increased with pleozyme treatment (Figure 3). Co-treatment with lipoic acid prolonged the effects of pleozyme-induced Nrf2 accumulation (Figure 4). In the context of a neurodegenerative disease, FRDA, pleozyme treatment also increased persulfidation of proteins (Figure 5).

## Data Availability

The original contributions presented in this study are included in the article/supplementary material. Further inquiries can be directed to the corresponding authors.

## Supplementary Materials

The following supporting information can be downloaded at https://www.mdpi.com/article/10.3390/nano14242007/s1. Figure S1. Pleozymes may not induce the aggregation of SOD1 in the presence of a sulfide donor. Changes in the fluorescence of the protein aggregation marker thioflavin T (40 µM) in the presence of 40 μM bovine superoxide dismutase 1 (SOD1) co-incubated with 150 μM disodium sulfide (Na_2_S), 150 µM Na_2_S, and 4 µg/mL pleozymes or with 150 µM hydrogen peroxide (H_2_O_2_). Increasing thioflavin T fluorescence with H_2_O_2_ treatment indicates the formation of SOD1 protein aggregates over time [75]. Lack of changes in fluorescence with Na_2_S treatment with and without pleozymes suggest that neither sulfide alone nor pleozymes in the presence of sulfides facilitate the pathological aggregation of SOD1 protein. Fluorescence readings (λ_Ex_./λ_Em_.: 420/480 nm) were measured every 15 min over a 72 h interval. n = 3 technical replicates (wells) per group. Shaded region: ±1 standard deviation. Figure S2. Persulfidation level of GAPDH. N = 4 technical replicates. Error bars represented standard deviation from 4 technical replicates.
