# Supplementary material for: Harshly Oxidized Activated Charcoal Enhances Protein Persulfidation with Implications for Neurodegeneration as Exemplified by Friedreich’s Ataxia
Source: Nanomaterials (Basel). 2024 Dec 13;14(24):2007. doi: 10.3390/nano14242007 (PMC11728766; doi:10.3390/nano14242007)
Supplement: Supplementary file 1 [file nanomaterials-14-02007-s001.zip › nanomaterials-3319217-supplementary.pdf]

Supplementary Figures

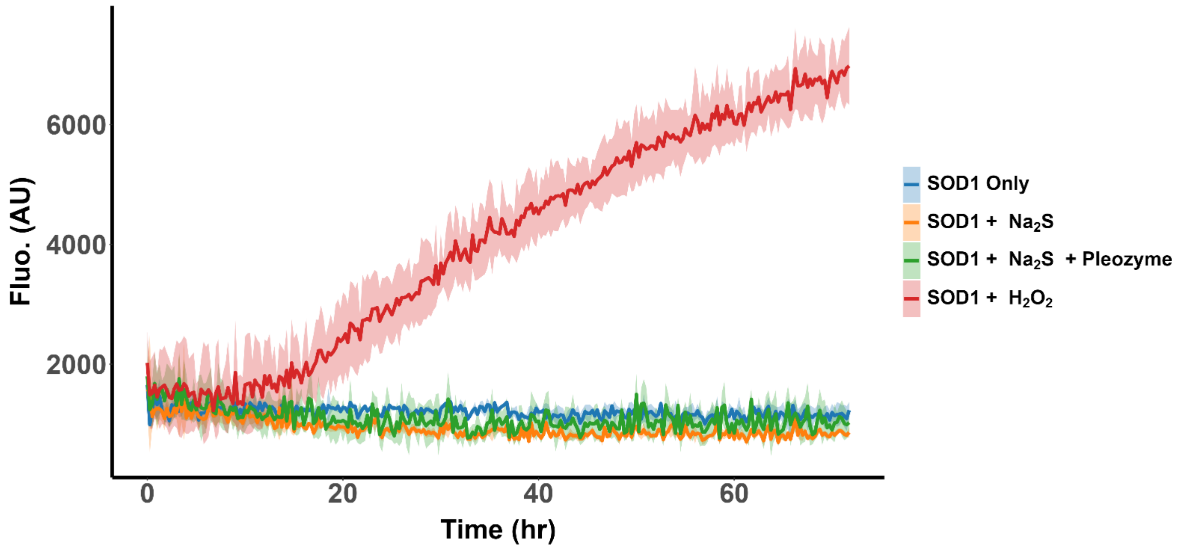

Figure S1. Pleozymes may not induce the aggregation of SOD1 in the presence of a sulfide donor. Changes in the fluorescence of the protein aggregation marker thioflavin T (40 μM) in the presence of 40 μM bovine superoxide dismutase 1 (SOD1) co-incubated with 150 μM disodium sulfide (Na_2S), 150 μM Na_2S, and 4 μg/mL pleozymes or with 150 μM hydrogen peroxide (H_2O_2). Increasing thioflavin T fluorescence with H_2O_2 treatment indicates the formation of SOD1 protein aggregates over time [75]. Lack of changes in fluorescence with Na_2S treatment with and without pleozymes suggest that neither sulfide alone nor pleozymes in the presence of sulfides facilitate the pathological aggregation of SOD1 protein. Fluorescence readings ($\lambda_{Ex.}/\lambda_{Em.}$: 420/480 nm) were measured every 15 min over a 72 h interval. n = 3 technical replicates (wells) per group. Shaded region: ±1 standard deviation.

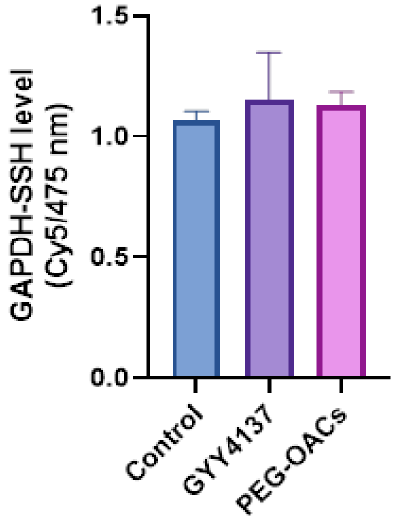

Figure S2. Persulfidation level of GAPDH. N=4 technical replicates. Error bars represented standard deviation from 4 technical replicates.
